# Synchronous metastatic differentiated high-grade thyroid carcinoma in lymph node from classic papillary thyroid carcinoma: a case report and literature review

**DOI:** 10.3389/fonc.2026.1790374

**Published:** 2026-05-25

**Authors:** Tuya E, Ya Zhang, Pin He, Xiaoxing Ye, Xieraili Wumener, Cong Liu, Ying Liang, Dehong Luo

**Affiliations:** 1 Department of Radiology, National Cancer Center/National Clinical Research Center for Cancer/Cancer Hospital & Shenzhen Hospital, Chinese Academy of Medical Sciences and Peking Union Medical College, Shenzhen, China; 2 Department of Pathology, National Cancer Center/National Clinical Research Center for Cancer/Cancer Hospital & Shenzhen Hospital, Chinese Academy of Medical Sciences and Peking Union Medical College, Shenzhen, China; 3 Department of Nuclear Medicine, National Cancer Center/National Clinical Research Center for Cancer/Cancer Hospital & Shenzhen Hospital, Chinese Academy of Medical Sciences and Peking Union Medical College, Shenzhen, China

**Keywords:** differentiated high-grade thyroid carcinoma, immunotherapy, papillary thyroid carcinoma, skip metastasis, targeted therapy

## Abstract

Differentiated high-grade thyroid carcinoma (DHGTC) is relatively uncommon. This case report described a 61-year-old male initially diagnosed with classic papillary thyroid carcinoma confined to the thyroid gland, accompanied by skip metastasis to a Level II lymph node. The metastatic lymph node exhibited histopathological progression to DHGTC. Following thyroidectomy for the primary tumor and neck dissection for the metastatic lymph node, the patient received adjuvant radiotherapy to the neck. Subsequently, pathological examination confirmed multiple pulmonary metastases (PMs) from DHGTC within six months of the initial diagnosis. Unfortunately, a post-therapeutic ¹³¹I scan revealed no uptake in the PMs. Genetic testing of the PMs revealed no mutations in BRAF, KRAS, or NRAS, and no RET rearrangement. The patient’s condition was subsequently managed with targeted therapy and immunotherapy, which achieved disease stabilization.

## Introduction

Differentiated high-grade thyroid carcinoma (DHGTC), a new entity in the 2022 World Health Organization (WHO) classification, is categorized along with poorly differentiated thyroid carcinomas (PDTC) as high-grade thyroid follicular cell-derived carcinomas (1, 2). DHGTC retains the architectural and cytologic features of their differentiated counterparts, such as papillary, follicular, or oncocytic components, and at least one of the following features: increased mitotic figures exceeding 5/2 mm^2^, or tumor necrosis (1, 2). This distinction is essential to separate DHGTC from PDTC, which displays insular, solid, and/or trabecular architectural patterns, the absence of papillary-like nuclear features, and at least one of the following features: convoluted nuclei, increased mitotic figures exceeding 3/2 mm^2^, or tumor necrosis (1, 2).

A recent meta-analysis reports a pooled DHGTC prevalence of 7.2% among well-differentiated thyroid carcinomas in clinically unselected patients, with aggressive subtypes exhibiting the higher risk (14.6%**–**16.8%), while the classic subtype papillary thyroid carcinoma (PTC) shows a significantly lower prevalence (4.2%) (3). The weighted mean age at initial diagnosis is 54.8 years (4). At initial diagnosis, the overall weighted average metastasis rate is 23.81%, the lymph node metastasis (LNM) rate is 42.23%, and extrathyroidal extension is 61.44% (4). The 5-year and 10-year disease specific survival (DSS) are 70%, 60% for DHGTC (5).

DHGTC usually manifests in one of three clinical scenarios (1): as a primary thyroid tumor (2), with synchronous distant metastases at initial diagnosis, or (3) as metachronous recurrence or distant metastasis during follow-up. In this context, we retrospectively analyzed the diagnostic process of this case with metastatic DHGTC and reviewed relevant literature to raise awareness of this disease.

## Case presentation

### Basic information

A 61-year-old man presented with an incidentally discovered mass in the left submandibular region that had persisted for over one month. He has a history of hypertension and has been regularly taking antihypertensive medications, maintaining good blood pressure control. No other significant medical history was noted.

### Physical examinations

Physical examination revealed a palpable mass in the left submandibular region, approximately 3 × 3cm in size, firm in consistency, with ill-defined borders, and moderately mobile. No cervical lymphadenopathy or thyroid abnormalities were noted.

### Laboratory findings

During hospitalization, the patient underwent a series of routine laboratory tests, including a complete blood count, glucose metabolism assessment, lipid profile, electrolyte panel, infectious disease screening (8-item panel), thyroid function panel (8 tests), liver and kidney function tests, Epstein-Barr virus serology, and carcinoembryonic antigen. The patient was diagnosed with diabetes and hyperlipidemia (hyperlipoproteinemia), while the other results showed no significant abnormalities.

### Imaging examination

**Figure 1:**
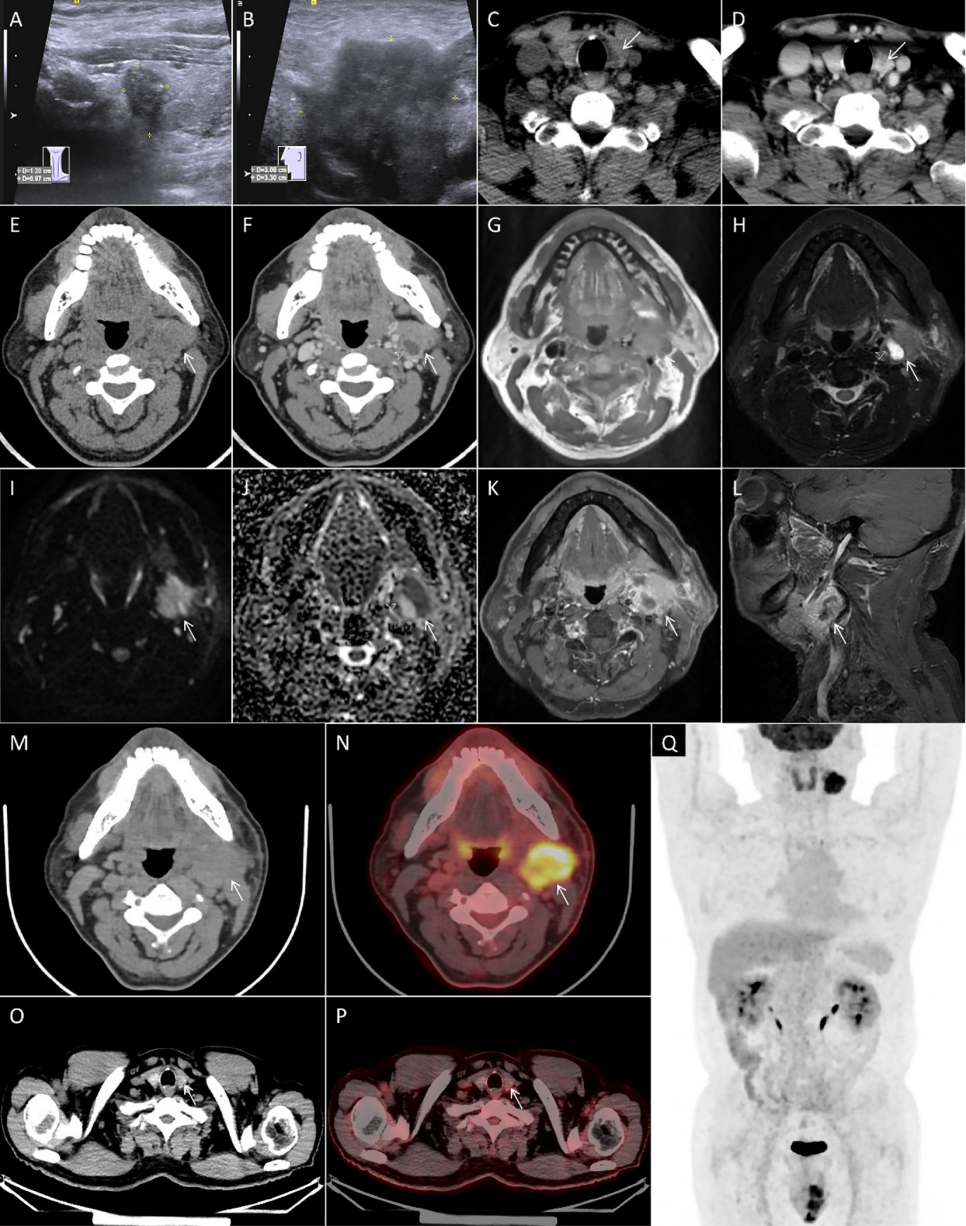
Results of the patient’s neck US, CT, MRI, and ^18^F-FDG PET/CT scans. US showed a 1.2 × 1.0cm hypoechoic nodule in the left thyroid upper pole **(A)**, classified as 4C according to the Chinese Thyroid Imaging Reporting and Data System (C-TIRADS) (6). US revealed a 3.3 × 3.1cm hypoechoic lesion in the left submandibular region **(B)**. CT demonstrated a suspected hypodense nodule in the left thyroid upper pole (white arrow in **(C, D)**). CT and MRI revealed an irregular left submandibular region mass (≈3.6 × 2.9 × 3.6cm), suspicious for malignancy (white arrow in **(E–L)**). PET/CT revealed intense uptake in the left submandibular region mass, with a maximum standardized uptake value (SUV_max_) of 8.0 (white arrow in **(M, N)**), and mild uptake in the thyroid nodule (SUV_max_ = 1.4) (white arrow in **(O, P)**), with no other foci of significantly increased uptake **(Q)**.

The patient underwent a comprehensive diagnostic workup during hospitalization. First, ultrasound (US) of the thyroid gland, submandibular glands, and cervical lymph nodes was performed. This was followed by contrast-enhanced computed tomography (CT) of the neck and chest, contrast-enhanced magnetic resonance imaging (MRI) of the neck, and whole-body ^18^F-fluorodeoxyglucose positron emission tomography/computed tomography (^18^F-FDG PET/CT). All imaging examinations presented are shown in **Figure 1**.

### Laryngopharyngology endoscopy

No abnormalities were found during the laryngopharyngological examination.

### Diagnosis, treatment, and follow-up

#### Primary diagnosis and treatment plan

Based on the comprehensive imaging and endoscopic examinations (**Figure 1**), the following lesions were identified in this patient (1): a mass suspicious for malignancy in the left submandibular region (2); suspected carcinoma in the upper lobe of the left thyroid; and (3) early-stage adenocarcinoma in the upper lobe of the left lung.

Simultaneous fine needle aspiration biopsy (FNA) of the left thyroid lobe nodule and the left submandibular region mass revealed (1): PTC in the left thyroid lobe, and (2) poorly differentiated carcinoma in the left submandibular region, likely salivary duct carcinoma. Immunohistochemistry (IHC) of the latter lesion showed AE1/AE3 (3+), CK7 (3+), calponin (–), P63 (–), P40 (rare +), AR (3+), HER2 (0), SMA (–), Ki-67 (40%), INI1 (+), and BRG1 (+). Based on a multidisciplinary team (MDT) consensus involving specialists in head and neck surgery, thoracic surgery, and radiology, the treatment plan included the simultaneous resection of the tumor in the left submandibular region and thyroid cancer, along with elective resection of the left upper lobe ground-glass opacity (GGO). The specific surgical plan for the neck was as follows (1): extended resection of the malignant tumor in the left submandibular region with left neck lymph node dissection (Levels I–IV), and (2) left thyroid lobectomy, isthmusectomy, and left Level VI lymph node dissection.

#### Primary surgery followed by adjuvant radiotherapy

**Figure 2:**
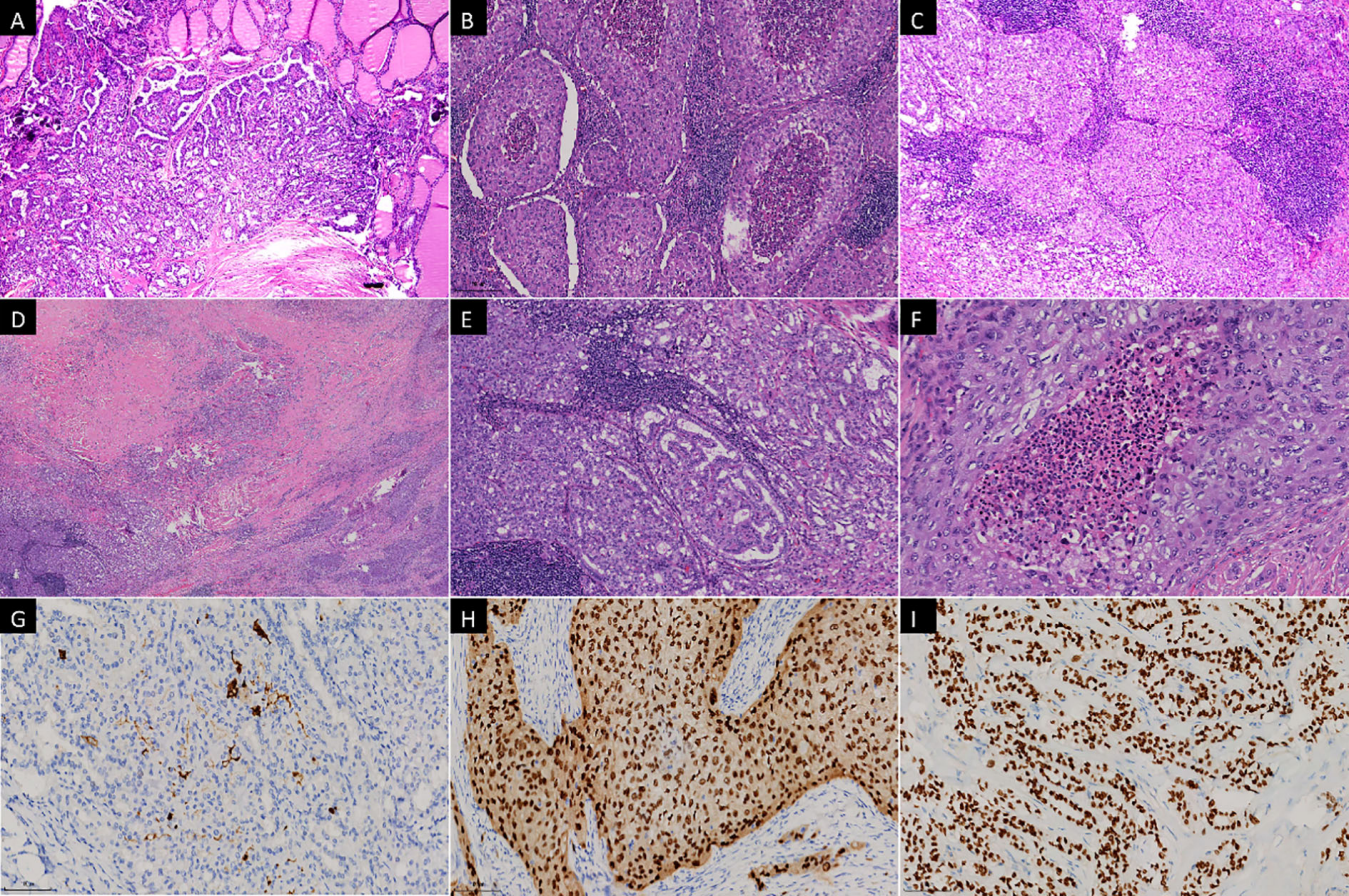
Results of the patient’s hematoxylin and eosin (HE) staining and immunohistochemistry (IHC). Postoperative pathological examination confirmed a 1.1cm classic-type PTC located in the left upper lobe, characterized by stromal fibrosis, capsular penetration into the perithyroidal fibrofatty tissue, and perineural invasion **(A)**. Pathological examination of the mass in the left submandibular region confirmed a metastatic, DHGTC within a level II lymph node, measuring 4.2cm in diameter **(B, C)**. The tumor involved the adjacent striated skeletal muscle and demonstrated angiolymphatic invasion. DHGTC met the diagnostic criteria, including tumor cells arranged in papillary and follicular patterns **(D, E)** and tumor necrosis **(D, F)**. IHC of the metastasis demonstrated rare positivity for thyroglobulin **(G)** and strong positivity for PAX8 **(H)** and TTF-1 **(I)**. Additional IHC markers were as follows: CK19 (3+), CK7 (3+), Ki-67 (30%+), GATA3 (–), GCDFP15 (–), AR (1+), HER2 (1+), CK5/6 (focal +), P40 (focal +), P63 (focal +), and calponin (–). The final pathological stage was pT1bN1b (Stage II) (7).

The surgical pathology results confirmed a PTC located in the upper pole of the left lobe of the thyroid gland, accompanied by interstitial fibrosis. The tumor measured 1.1cm in diameter and involved the thyroid capsule as well as the surrounding fibrofatty tissue. Nerve invasion was also evident (**Figure 2A**). The mass in the left submandibular region was identified as DHGTC, exhibiting the extranodal extension (ENE) with further invasion into the adjacent skeletal muscle and demonstrating angiolymphatic invasion (**Figures 2B–F**). Dissection of the left cervical lymph nodes (Levels I**–**IV and VI) revealed metastatic carcinoma in 1 of 36 lymph nodes. Pathological examination confirmed classic PTC with metastasis to a Level II lymph node in the left neck, where the metastatic lesion had progressed to DHGTC. In summary, based on the 8th edition of the American Joint Committee on Cancer (AJCC) staging system, the final pathological stage was pT1bN1b (Stage II) (7). This case was designated as high-risk in the initial recurrence risk stratification (8). Following the MDT discussion, adjuvant external beam radiation therapy was administered. The radiation dose for the metastatic lymph node area in the left cervical level II region was 66 Gy, delivered in 2 Gy fractions over 33 sessions. For the left thyroid lobe tumor bed and the left cervical levels Ib, II, III, and IVa drainage areas, the radiation dose was 60.06 Gy, delivered in 1.82 Gy fractions over 33 sessions.

#### Thoracic surgery for primary lung cancer and pulmonary metastases

**Figure 3:**
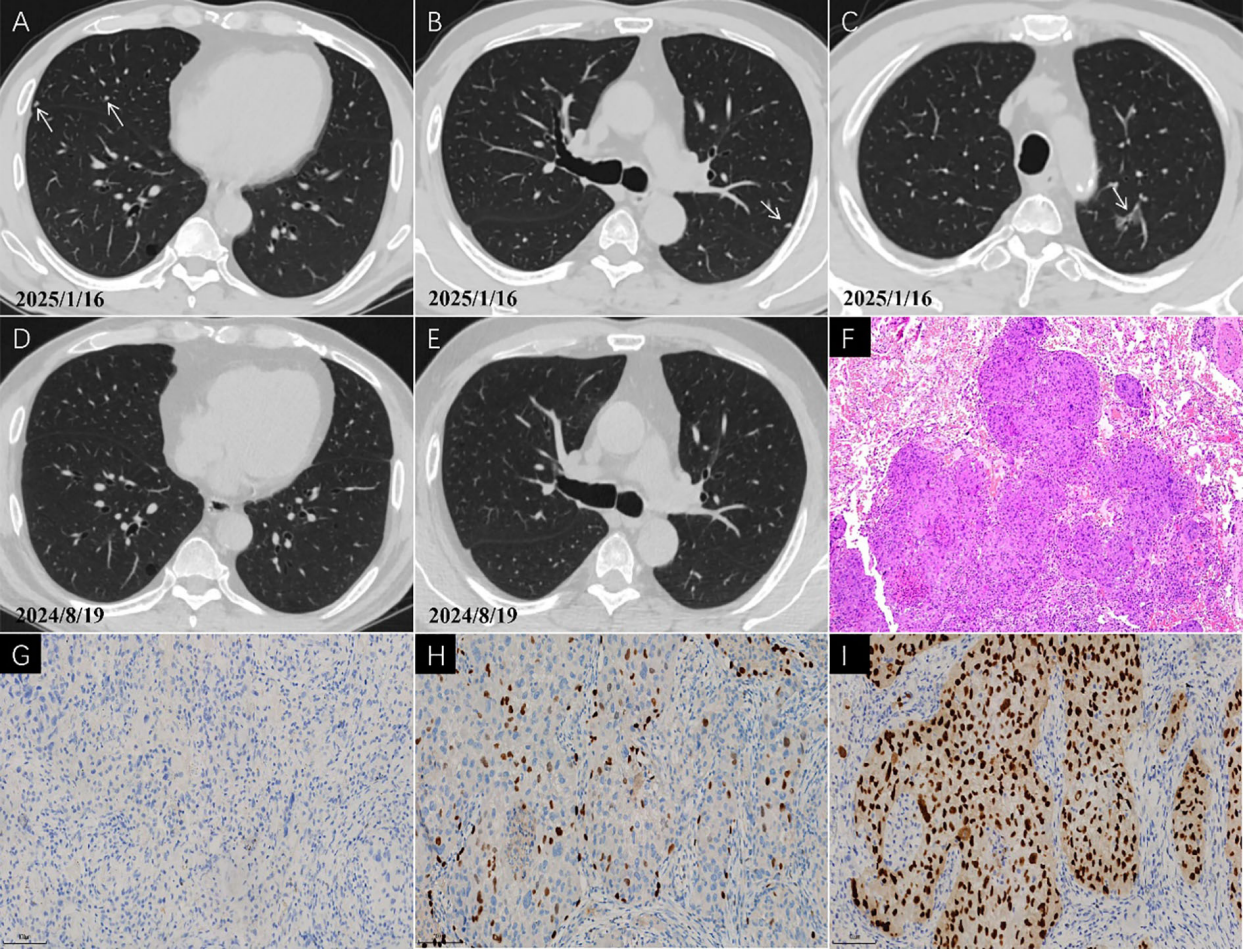
Results of the patient’s chest CT scans, HE staining, and IHC. Approximately five months after the preoperative chest CT scans **(D, E)**, follow-up CT **(A–C)** revealed newly developed multiple solid micronodules in both lungs, ranging from 2 to 5mm in diameter, highly suspicious for PMs from thyroid carcinoma. However, the GGO in the left upper lobe remained stable (white arrow in **(C)**). Surgical pathology later confirmed intrapulmonary metastasis of DHGTC (**F**). IHC showed negativity for thyroglobulin **(G)** and positivity for TTF-1 **(H)** and PAX8 **(I)**. Additional IHC markers were as follows: CK7 (3+), CK19 (3+), Napsin A (–), AR (1+), and GCDFP-15 (–).

Approximately five months after the cervical surgery, a preoperative chest CT was performed in preparation for the resection of GGO in the left upper lobe. Compared to the initial chest CT scan taken approximately five months earlier, the GGO remained stable; however, several new, randomly distributed solid micronodules (diameter range: 2–5 mm) were observed. These newly identified nodules were suspected to be metastases from thyroid carcinoma (**Figures 3A, B**). Ultimately, segmentectomy of the left upper lobe was performed. Pathological examination revealed that the GGO was a well-differentiated adenocarcinoma (pT1bN0). Additionally, pathological examination confirmed multiple pulmonary metastases (PMs) from DHGTC, measuring 1 to 5mm in diameter (**Figures 3F–I**). At this juncture, following disease progression, the pathological stage was upstaged pT1bN1bM1, Stage IVB (7).

#### Radioactive iodine therapy and systemic treatment

**Figure 4:**
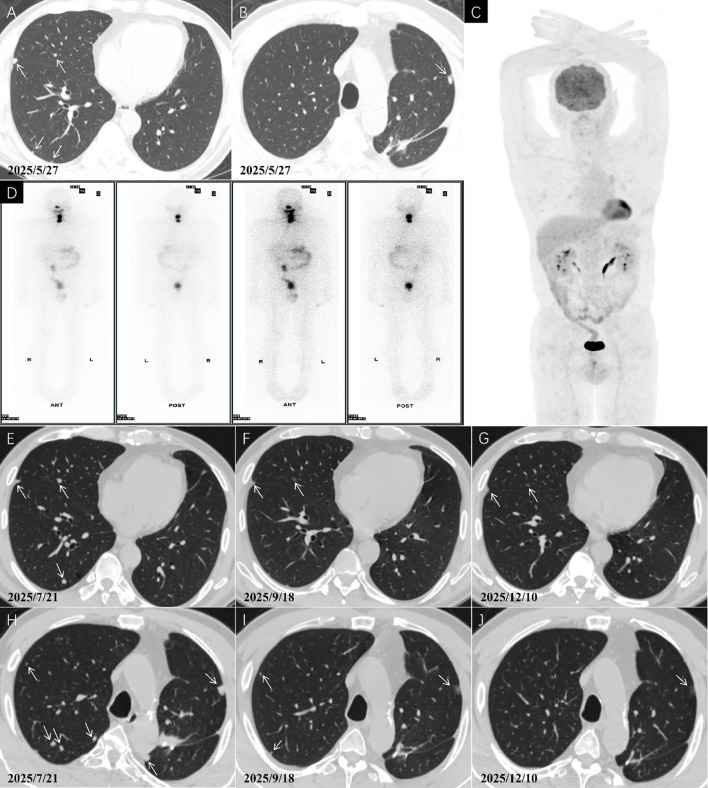
Results of the patient’s ^18^F-FDG PET/CT, post-therapeutic (200mCi ^131^I) whole-body scan (WBS), and follow-up chest CT scans. The patient’s initial post-operative PET/CT scan revealed progression of multiple PMs (white arrow in **(A, B)**), with the largest measuring approximately 8.0mm in diameter (white arrow in **(B)**), and no evidence of additional metastatic disease **(C)**. Subsequently, a therapeutic dose of ^131^I showed no uptake in these PMs **(D)**. Follow-up chest CT scans conducted before and after the initiation of targeted combined with immunotherapy demonstrated further progression (white arrow in **(E, H)**) compared to the pre-radioiodine therapy scan (white arrow in **(A, B)**), with the largest lesion increasing to approximately 10.4mm (white arrow in **(H)**). However, subsequent evaluations following systemic therapy indicated a positive treatment response, with the PMs decreasing in both size and number (white arrow in **(F, G, I, J)**). The largest lesions measured approximately 8.1mm and 7.8mm on successive scans (white arrow in **(I, J)**).

**Figure 5:**
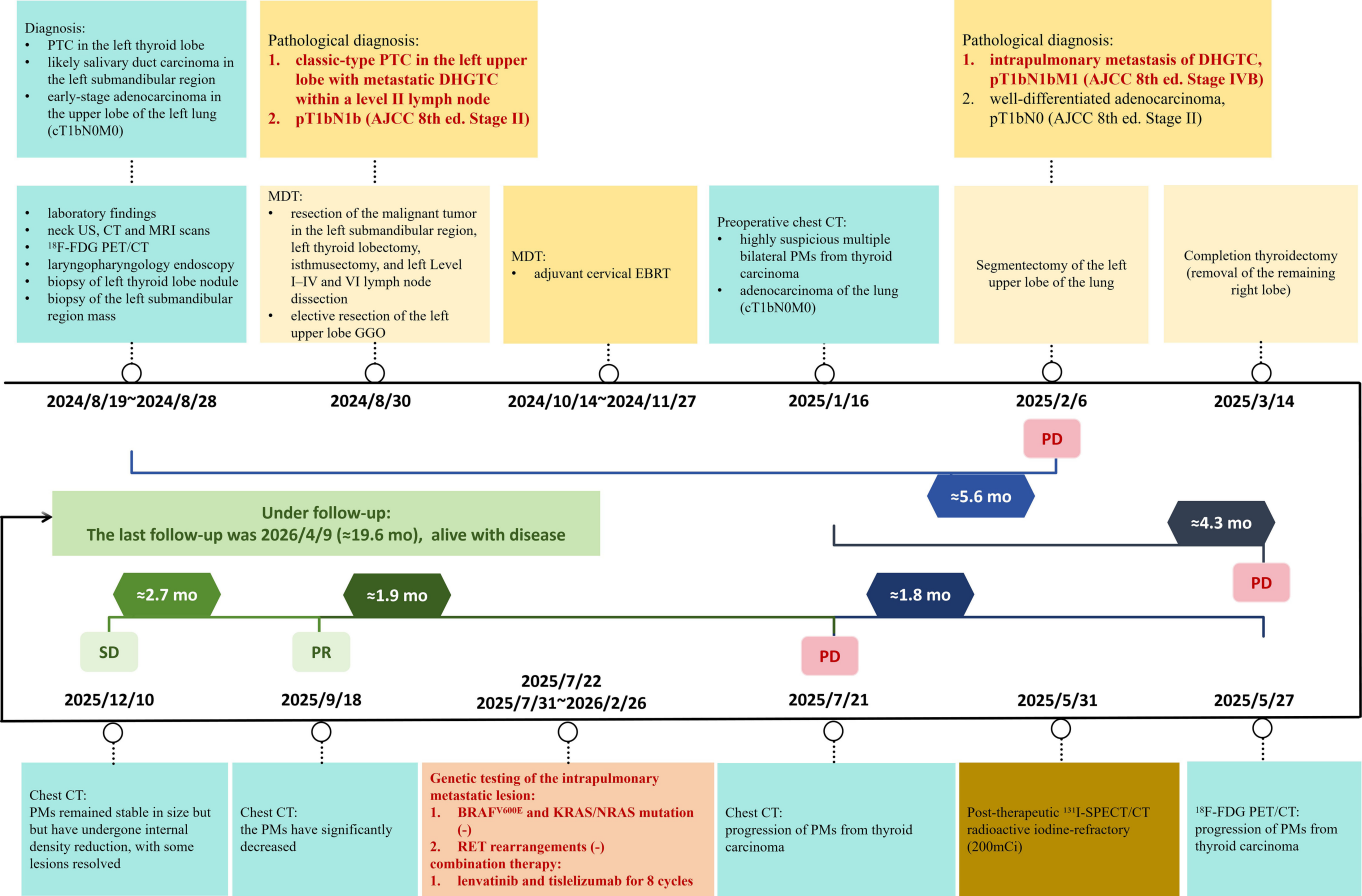
Timeline of diagnosis, treatment and response assessments. PTC, papillary thyroid carcinoma; DHGTC, differentiated high-grade thyroid carcinoma; MDT, multidisciplinary team; GGO, ground-glass opacity; EBRT, external beam radiation therapy; PMs, pulmonary metastases; PD, progressive disease; PR, partial response; SD, stable disease; SPECT, single photon emission computed tomography. mo, month. Radiological assessments were performed according to response evaluation criteria in solid tumors (RECIST v1.1) (11).

In preparation for radioactive iodine therapy, the patient underwent the first PET/CT evaluation approximately 4 months after thoracic surgery. Compared to the preoperative chest CT, this assessment revealed progression of the PMs (**Figures 4A–C**). Unfortunately, a post-therapeutic (200mCi ^131^I) scan revealed no uptake in the PMs (**Figure 4D**), indicating radioiodine-refractory differentiated thyroid carcinoma (RAIR-DTC) (9). Approximately 2 months after from postoperative PET/CT scan, a chest CT showed further progression of the PMs (**Figures 4E, H**). Genetic testing was performed to detect mutations in BRAF^V600E^ and KRAS/NRAS, as well as RET rearrangements in the PMs. All the results were negative. Consequently, the patient’s subsequent systemic treatment regimen consisted of targeted therapy combined with immunotherapy, specifically lenvatinib and tislelizumab (10). Two follow-up chest CT scans conducted after initiating this systemic therapy demonstrated treatment efficacy (**Figures 4F, G, I, J**). Currently, the patient is alive with disease on ongoing therapy. The complete timeline of diagnosis, treatment, and response assessment is summarized in **Figure 5**.

## Discussion

To summarize this case briefly, the patient had a small PTC in the left upper lobe (pT1b), accompanied by a significantly larger metastatic deposit in a left Level II lymph node (pN1b), which progressed to DHGTC. Subsequently, PMs developed within a short interval of approximately six months, and the histological type was also DHGTC (pM1). Following the failure of radioiodine therapy, systemic therapy was initiated and proved effective.

### Skip metastasis in papillary thyroid carcinoma: re-evaluating the prognosis

Metastatic spread in PTC characteristically involves the central compartment lymph nodes (Level VI), progressing from the ipsilateral to the contralateral side before extending to the lateral cervical compartments (Levels II**–**V). However, the presence of lateral lymph node metastasis (LLNM) without evidence of central compartment involvement is defined as skip LNM. The rate of skip metastasis is about 5%**–**25% (12). Thyroid capsular invasion, multifocality, tumor size (>1cm), and upper lobe location are significantly associated with skip metastasis (13). This case meets three of the four high-risk factors, excluding multifocality. Anatomically, the upper pole of the thyroid gland is closer to the lateral cervical lymph nodes than to the central compartment lymph nodes. This proximity may enable tumors located in the upper pole to bypass the central lymph nodes and directly metastasize to the lateral cervical lymph nodes along the superior thyroid blood vessel (12). As previously reported, patients with PTC located in the upper portion are more likely to experience tumor recurrence than those with tumors located in other subregions (14).

Some studies have reported that the rates of LLNM at initial diagnosis for DHGTC with primary foci located within the thyroid gland are 16% (41/252), 50% (7/14) and 78% (14/18) (15–17); however, they did not specify whether these were skip metastases. Therefore, the true incidence and clinical characteristics of skip metastasis in DHGTC remain largely unknown. Moreover, high-grade transformation occurring synchronously within a metastatic focus is exceedingly rare. Our case demonstrates DHGTC arising not *de novo*, but from a PTC skip metastasis that underwent such transformation within the lymph node. This confluence of a skip metastasis pattern and synchronous metastatic-site transformation underscores the exceptional rarity and instructive value of this case.

The current literature indicates that in a long-term study (average of 82 months) of T1-stage PTC, the recurrence rate in the skip N1b (sN1b) group is 11.3% (14/124), demonstrating that sN1b itself is not an independent prognostic factor for worse recurrence-free survival, with outcomes comparable to those of N1a and N1b disease (18). However, this favorable prognosis cannot be extrapolated to high-grade subtypes. In stark contrast, our present case of DHGTC—despite sharing a similar initial stage (pT1aN1b)—reveals a distinctly different clinical course, characterized by rapid progression to PMs within six months. This aggressive behavior is biologically explained by the Ki-67 index of 30% and angiolymphatic invasion in the metastatic focus, aligning with the highly proliferative nature and poor prognosis typical of DHGTC (1, 5, 16, 19, 20). This discrepancy underscores a critical point: while skip metastasis in PTC is generally associated with a favorable prognosis comparable to other N1 stages, our case suggests that in DHGTC, it may signal a high risk for rapid distant spread and a consequently poorer prognosis, necessitating vigilant surveillance and consideration of systemic therapy.

### High-grade transformation within the metastatic niche

The most challenging aspect of this case was the histological discrepancy between the metastatic lymph node and the primary tumor during the initial surgery. The IHC of the LNM showed only focal positivity for thyroglobulin (TG), whereas subsequent PMs demonstrated a complete loss of TG expression. This progression indicates the tumor’s progressive dedifferentiation during metastatic evolution. In terms of molecular characteristics, DHGTC generally arises from differentiated thyroid cancer through multistep genetic evolution. Early mutations primarily involve the BRAF or RAS genes, and as the tumor progresses, secondary invasive mutations such as TERT and TP53 emerge (1, 21). These mutations collectively drive dedifferentiation, metastasis, and treatment resistance (1, 21). In the case we reported here, the patient was tested only for common genes, specifically BRAF^V600E^, KRAS/NRAS and RET, and no mutations or rearrangements were detected. However, as noted, DHGTC is characterized by aggressive dedifferentiation, often driven by additional genetic alterations such as TERT promoter mutations, TP53/PIK3CA mutations, ALK/NTRK rearrangements (1, 21). Furthermore, a high tumor mutation burden (TMB) is also a relevant genomic feature associated with such advanced disease. A Key limitation of this study is the absence of data on these critical markers. Assessing them in future investigations will be essential to fully elucidate the molecular profile and potential therapeutic vulnerabilities of this tumor.

### Literature synthesis and institutional cohort comparison

To contextualize the rarity and novelty of this case, we analyzed our institutional experience and reviewed the relevant literature. To date, we have treated four DHGTC cases. Two patients developed distant metastasis: one with synchronous lymph node and lung involvement (the current case), and another with metachronous pleural metastasis developing after an interval of approximately 79.3 months, characterized by a RET rearrangement and wild-type BRAF ^V600E^. Both patients remain alive with disease at 14 and 18.8 months, respectively. The remaining two cases presented with localized intrathyroidal disease. Specifically, the patient with pT1N0 achieved no evidence of disease for 38.6 months, while the patient with pT3N1b was lost to follow-up. Pooled cohort data indicating a 5-year DSS of approximately 70% for DHGTC (5). Additionally, a recent study reported three DHGTC cases that developed PMs after 42–125 months of follow-up, showing RET rearrangements or BRAF ^V600E^/TERT mutations (17), the present case stands out. Notwithstanding a less advanced initial T stage (pT1aN1b vs. pT3bN1b), our patient developed distant metastasis within a remarkably shorter interval (6 month) compared with 42–125 months reported in the literature (17). This constellation of features—intranodal dedifferentiation combined with explosive metastatic potential—underscores the exceptional rarity and clinical significance of this case.

### Therapeutic efficacy

A study reported that male gender, age over 45 years, and dedifferentiation are independent predictors of mortality in patients with metastatic differentiated thyroid cancer, and that traditional treatments are often ineffective (22). In line with previous studies, our case also exhibited RAI-refractory thyroid carcinoma. Currently, targeted therapy and immunotherapy have emerged as promising options for advanced metastatic thyroid cancer (23). Although no effective driver gene mutations were identified in this patient to guide precise targeted drug selection, lenvatinib remains a first-line systemic treatment option for RAIR-DTC (10). The application of immunotherapy in RAIR-DTC remains exploratory. Previous studies have reported that multikinase inhibitors (MKIs) and immunotherapy can work synergistically against tumors. Lenvatinib could modulates the immune microenvironment, thereby enhancing the efficacy of programmed death-1(PD-1) inhibitors. One study confirmed that in patients with RAIR-DTC, the combination of lenvatinib and pembrolizumab (also a PD-1 inhibitor) achieved a high objective response rate (ORR 65.5%) and prolonged progression-free survival (PFS 26.8 months) in treatment-naïve to multikinase inhibitors, and also demonstrated clear efficacy in lenvatinib-resistant patients (ORR 16%, median PFS 10.0 months) (24). The same study indicated that programmed death-ligand 1(PD-L1) expression levels showed no significant correlation with treatment response to lenvatinib plus pembrolizumab (24). Although that trial used pembrolizumab, its mechanism of action is the same as that of tislelizumab. In addition, several studies have confirmed the safety and efficacy of the combination of lenvatinib and tislelizumab in other solid tumors such as hepatocellular carcinoma and biliary tract cancer (25, 26). Notably, real-world case reports confirm durable responses to tislelizumab in aggressive thyroid carcinoma, reinforcing this therapeutic rationale (27, 28). The present patient benefited from the combination therapy, with PMs markedly shrinking and decreasing after only two months of treatment. As research on the tumor immune microenvironment deepens, immunotherapy is likely to become an important complementary approach in the future management of thyroid cancer.

## Conclusion

In conclusion, we describe a case of DHGTC identified in skip LNM of classic PTC, with rapid progression to PMs within six months of the initial diagnosis. The mass in the left submandibular region was initially suspected to be a primary salivary gland tumor, metastatic squamous cell carcinoma from an unknown primary (e.g., tonsillar cancer), or lymphoma. Contrary to these expectations, pathological examination revealed DHGTC. As mentioned above, although skip metastasis can occur in PTC, the occurrence of pathological upstaging via this mechanism was not anticipated in our initial differential diagnosis.​ This case highlights a potential gap in the evaluation of skip lymph nodes in PTC. Clinically, it underscores the necessity of considering high-grade transformation even in anatomically unexpected nodal stations. Furthermore, this case illustrates that in patients with rapidly progressive, RAI-refractory DHGTC, conventional therapeutic options were limited. Although tislelizumab is not yet guideline-recommended, the combination therapy of lenvatinib and tislelizumab was empirically initiated based on the patient’s clinical trajectory and preference, resulting in significant radiographic response and disease control—highlighting the potential value of personalized, mechanism-driven therapeutic strategies in refractory high-grade thyroid malignancies when standard approaches fail.

## Data Availability

The original contributions presented in the study are included in the article/supplementary material. Further inquiries can be directed to the corresponding authors.
